# Role of Immuno-Inflammatory Signals in Liver Ischemia-Reperfusion Injury

**DOI:** 10.3390/cells11142222

**Published:** 2022-07-17

**Authors:** Christof Kaltenmeier, Ronghua Wang, Brandon Popp, David Geller, Samer Tohme, Hamza O. Yazdani

**Affiliations:** 1 Department of Surgery, University of Pittsburgh Medical Center, Pittsburgh, PA 15213, USA; kaltenmeierct2@upmc.edu (C.K.); ronghuaw@upmc.edu (R.W.); gellerda@upmc.edu (D.G.); tohmest@upmc.edu (S.T.); 2 Lake Erie College of Osteopathic Medicine, Erie, PA 16509, USA; bpopp36689@med.lecom.edu

**Keywords:** ischemia-reperfusion injury, ROS, Kupffer cells, neutrophils, NETs, DAMPs, platelets, thrombosis, miRNA

## Abstract

Ischemia reperfusion injury (IRI) is a major obstacle in liver resection and liver transplantation. The initial step of IRI is mediated through ischemia which promotes the production of reactive oxygen species in Kupffer cells. This furthermore promotes the activation of pro-inflammatory signaling cascades, including tumor necrosis factor-alpha, IL-6, interferon, inducible nitric oxide synthase, TLR9/nuclear-factor kappa B pathway, and the production of damage-associated molecular patterns (DAMPs), such as ATP, histone, high mobility group box 1 (HMGB1), urate, mitochondrial formyl peptides and S100 proteins. With ongoing cell death of hepatocytes during the ischemic phase, DAMPs are built up and released into the circulation upon reperfusion. This promotes a cytokines/chemokine storm that attracts neutrophils and other immune cells to the site of tissue injury. The effect of IRI is further aggravated by the release of cytokines and chemokines, such as epithelial neutrophil activating protein (CXCL5), KC (CXCL1) and MIP-2 (CXCL2), the complement proteins C3a and C5a, mitochondrial-derived formyl peptides, leukotriene B4 and neutrophil extracellular traps (NETs) from migrating neutrophils. These NETs can also activate platelets and form Neutrophil-platelet microthrombi to further worsen ischemia in the liver. In this review we aim to summarize the current knowledge of mediators that promote liver IRI, and we will discuss the role of neutrophils and neutrophil extracellular traps in mediating IRI.

## 1. Introduction

**Figure 1:**
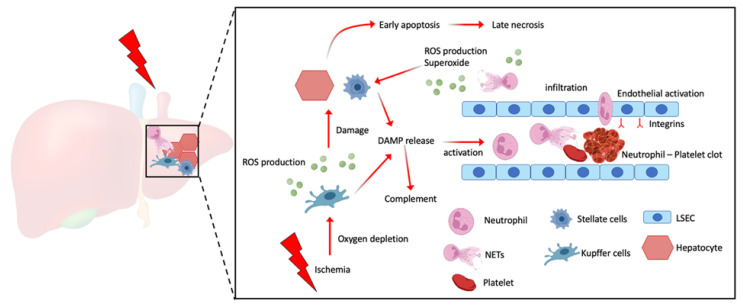
Schematic illustration showing prolonged ischemia reduces hepatic tissue oxygenation resulting in increased ROS production and DAMP release by both Kupffer cells and hepatic stellate cells which can induce hepatic cell death. These DAMPs can play a role as an innate immune cell attractant and activator, especially neutrophils to undergo NETosis. NETs can further promote platelet activation and capture resulting in microthrombi.

Liver ischemia and reperfusion injury is a frequent consequence in a variety of surgical procedures, and it is well established that IRI is a major cause of morbidity and mortality in liver resection and transplantation surgery [1,2,3]. Prolonged IRI reduces tissue oxygenation resulting in the depletion of adenosine triphosphate (ATP) and subsequent transition to anaerobic metabolism in hepatic resident cells. During transplantation, prolonged ischemic time is associated with delayed graft function, early rejection and increased risk of complications [4]. The pathogenesis of IRI is fueled by several different mechanisms such as oxidative stress, inflammatory responses, cell death, Kupffer cell activation, Neutrophil migration and activation. In general, it is known that ongoing ischemia promotes the build-up of waste products as several cellular functions fail to maintain homeostasis under oxygen deprivation. Cells start producing reactive oxygen species (ROS) that increase DNA and organelle damage. Prolonged ischemia increases the production of damage-associated molecular patterns (DAMPs) that are released into the surrounding tissue upon cell death [5]. These DAMPs can increase pro-inflammatory signaling cascades in surrounding cells as well as activate the complement system (Figure 1). With restoration of blood flow, several pathways are initiated that further promote cellular damage. DAMPs are flushed into the circulation and thereby promote the activation and recruitment of immune cells to the site of tissue injury [6,7]. Following reperfusion, neutrophils are among the first cells to enter the liver and there is overwhelming evidence that excessive neutrophil infiltration contributes to the pathogenesis of IRI. In this review, we will discuss molecular mechanisms of ischemia reperfusion injury and the current understanding of the role of neutrophils in mediating IRI.

## 2. Reactive Oxygen Production and Its Regulation during Ischemia

During the initial stage of IR (within 2h after reperfusion), Kupffer cells are the main producers of reactive oxygen species (ROS) and other pro-inflammatory mediators including tumor necrosis factor TNF-α and interleukin (IL)-6 [8]. ROS production can promote lipid peroxidation and increase cell membrane and mitochondrial electron leakage. Furthermore, ROS increases signal transduction pathways, caspase activation and promotes cell death of hepatic stellate, Kupffer and endothelial cells [9].

Depletion of intracellular ATP during ischemia of the liver happens within seconds, with ongoing ischemic stress, liver cells are unable to produce ATP to maintain cellular metabolism. With the lack of oxygen cells switch toward anaerobic metabolism. During oxygen-rich states, mitochondria containing cells can use pyruvate to enter the citric acid cycle and undergo oxidative phosphorylation, a process that cannot take place in the absence of oxygen. In oxygen-deprived tissue, pyruvate remains within the cytoplasm and converts to lactate, a process known as anaerobic glycolysis. With lack of oxygen the overall net production is only 2 ATP per glucose molecule compared to 32 ATP per glucose molecule during oxidative phosphorylation.

Reperfusion leads to a dramatic rise in oxygen delivery which exceeds the rate at which cellular metabolism is capable of working, thereby producing free radicals that promote DNA and cellular damage. In fact, the main driver of ROS production during IRI is the massive accumulation of succinate within the mitochondria. Upon reperfusion, succinate is oxidized within the electron transport chain and ROS is produced [10]. Other producers of ROS include the conversion of xanthine dehydrogenase to xanthine oxidase, nicotinamide adenine dinucleotide phosphate (NADPH) oxidase activation and uncoupling of the mitochondrial electron transport chain as described earlier.

One way to regulate ROS production is through the phosphatidylinositol-3-kinase/protein kinase B (PI3K/Akt) signaling pathway. The PI3K/AKT pathway is involved in various cellular physiological and pathological functions. This pathway is activated during the early phase of IRI where it plays a role in anti-apoptosis, -inflammation and -oxidation. PI3K is a well-studied redox sensitive kinase that is activated in an acidic environment where increased levels of ROS can be found. The activated PI3K/Akt signaling pathway promotes the generation of heme-oxygenase-1 (HO-1) by upregulating the expression of Nrf2 which decreases the oxidative stress following IRI [8,9].

## 3. Cell Death Signaling during IRI

During the ischemic phase of IRI, the two predominant cell populations that are injured are hepatocytes and sinusoidal endothelial cells (SECs). Apoptosis and necrosis are both seen during IRI depending on the duration of ischemia and time of reperfusion. The hallmarks of apoptosis include shrinkage of the cell, chromatin condensation, nuclear fragmentation and the formation of apoptotic bodies. Necrosis can be characterized by the presence of mitochondrial and cell swelling with a loss of plasma membrane integrity and leakage [11]. Interestingly, the intensity of a given stimulus can have varying effects and can promote the activation of apoptotic or necrotic pathways. For example, peroxynitrite, an ROS, can promote apoptosis at low concentrations; however, it can also lead to necrotic cell death once intracellular ATP levels are fully depleted [12,13]. Several studies have shown the presence of apoptotic hepatocytes following IRI by using TUNEL staining. However, these studies have also shown that despite the presence of apoptotic pathways in hepatocytes, the final form of cell death following prolonged ischemia and reperfusion remains necrosis [14,15]. Apoptosis is an extremely complex pathway with a close relation to free radical production, intracellular calcium overload, cytokine activation, caspase and B lymphocyte tumor-2 (Bcl-2) gene expression. When cells receive apoptosis signal stimulations, the Bcl-2 protein regulates the permeability of the mitochondrial membrane. Apoptosis leads to irreversible opening of the mitochondrial permeability transition pore (mPTP), which leads to the release of cytochrome C into the cytoplasm. Within the cytoplasm, cytochrome C interacts with Apoptosis protein activated factor 1 (Apaf-1) which activates the caspase 9 precursor though an auto-cleavage process. This complex together with caspase 3 can further induce apoptotic cell death [16,17]. Activated PI3K/Akt signaling pathway inhibits caspase 3-mediated cell death by phosphorylating Bcl-2/Bcl-XL-associated death promoter (Bad). Studies have demonstrated that activation of the PI3K/Akt/mTOR protects cells from apoptosis and significantly improves IRI [18,19,20]. Following tissue damage, chemokines are locally secreted by parenchymal cells to create a gradient to help in the migration of neutrophils from the blood. It has been demonstrated that following liver IRI, the expression of CXCL1 and CXCL2 can increase 100-fold in the ischemic lobes. This observation goes along with the finding of expression of these chemokines on the luminal surface of liver sinusoids in necrotic areas. Studies have shown that CXCL2 increases during the early phase of reperfusion prior to the detection of neutrophils and may therefore be involved in the initial recruitment [5].

## 4. Factors Contributing to Damage following Reperfusion

In addition to the inflammation and damage caused by apoptosis and necrosis, the reperfusion phase can further exacerbate inflammation. This is in part due to the delivery of blood, immune cells and oxygen into previously ischemic tissue thereby leading to activation of toll-like receptor 4 (TLR4) and complement signaling that promote further tissue damage. As oxygen is re-introduced into previously hypoxic and ischemic environments, it overwhelms metabolic pathways of damaged cells, and further promote production and release of ROS. Sudden oxygen influx causes uncoupling of the electron transport chain in mitochondria, further preventing ATP generation. Ischemic cells lack antioxidants, which are normally present in a healthy oxygenated state and function to prevent oxidative damage. In these starving cells, oxygen reacts with ATP degradation products, such as adenosine and xanthine that had previously accumulated in cells undergoing necrosis [21]. This interaction causes the generation of ROS, which include superoxide, hydrogen peroxide and reactive nitrogen species [22]. It has further been shown that TLR4 expression on non-parenchymal liver cells, such as Kupffer cells and SECs, is necessary for recognition of ischemic cells and activation of TLR4-mediated liver IRI. As previously mentioned, TLR4 activation leads to downstream activation of JNK and NF-KB signaling pathways, which lead to the activation and gene transcription of inflammatory genes [23]. JNK is a subgroup of the MAPK family that is specifically activated via TLR4 signaling and has been found to be present in the liver after IRI [24]. Tsung et al. have shown that mice lacking TLR4 demonstrated decreased levels of IRI. In addition, they showed that TLR4 ko mice had decreased levels of JNK and NF-KB signaling, suggesting that activation of TLR4 is responsible for promoting downstream proinflammatory signaling in the liver after the reperfusion phase of IRI [25].

As previously mentioned, the activation of the complement system is an important contributor to inflammation seen in liver reperfusion after ischemia. The complement system is part of the innate immune system and can be activated by one of three pathways. These pathways include the classical pathway, which is antibody dependent, the alternative pathway and the mannose-binding lectin pathway [22]. In IRI, the complement cascade is activated by detecting cellular contents that were released into the extracellular space as a result of ischemia. Complement activation leads to the formation of the soluble bioactive peptides, C3a and C5a, and the membrane attack complex, which results in the recruitment of inflammatory cells [26].

The anaphylatoxins C3a and C5a promote the accumulation and infiltration of PMNs, induce smooth muscle contraction, increase vascular permeability, increase the release of histamine and stimulate Kupffer cell activation, which produces TNF-alpha, IL-1 and ROS [27]. The ongoing release of pro-inflammatory chemokines and cytokines further promotes and increases the subsequent neutrophil-mediated inflammatory response.

## 5. Neutrophil Trafficking via Cellular Adhesion Molecules and Chemokine Signaling

Under hypoxic stress, Kupffer cells increase the production of DAMPs such as high-mobility group B1 (HMGB1), S100, heat shock proteins and circulating DNA/RNA that can all trigger a variety of inflammatory immune responses. HMGB1 can activate Toll-like receptor 4 (TLR4) which in turn triggers the recruitment of MyD88 promoting several downstream signaling pathways leading ultimately to the production of NF-κB, TNF-α and IL-6 which promote the upregulation of adhesion molecules on the luminal site of endothelial cells to aid with migration of neutrophils. Studies have shown that PI3K can inhibit the activation of the forementioned pathway and thereby alleviate IRI [28,29]. Shen et al. have shown that the activation of the PI3K/Akt signaling pathway lead to increased expression of anti-inflammatory cytokines IL-4 and IL-10 and decreased expression of TNF-α, IL-6 and IL-1β to ultimately reduce the IRI-induced liver inflammatory response [30].

Following the reperfusion of blood into the previously ischemic tissues, neutrophils are one of the first cell types that are activated via circulating DAMPs such as HMGB1 and ATP. As neutrophils are called to the area of inflammation, they adhere to the endothelial wall and transmigrate into the damaged tissue. Weibel-Palade bodies, which are produced by endothelial cells, secrete P-selectin, an important chemokine in neutrophil trafficking. P-selectin increases endothelial cell permeability which in turn slows down neutrophils, as they travel through the bloodstream, and allows them to roll along the endothelial cell wall [5]. This slowed neutrophil transit allows intercellular adhesion molecule (ICAM)-1 to promote adhesion of the neutrophil to the endothelial cell wall. ICAM-1 binds to integrins, which are expressed on the cell surface of activated neutrophils and causes neutrophils to adhere tightly to the blood vessel wall. From there, neutrophils transmigrate across the endothelium and travel towards chemokines produced by damaged tissues as previously described [5].

With P-selectin and ICAM-1 serving as mediators of the initial stage of neutrophil transmigration into damaged tissue, it has previously been hypothesized that the removal of P-selectin and ICAM-1 in experimental models would lead to decreased neutrophil-mediated IRI [31]. The dependence of neutrophil infiltration on cellular adhesion molecules appears to be inconsistent as some studies have found neutrophil infiltration to be dependent on cellular adhesion molecules, whereas others have found neutrophil infiltration to be independent of cellular adhesion molecules. In one experiment, Monson et al. compared wild type mice and P-selectin/ICAM-1 deficient mice (P/I knockout mice), with the aim of assessing the role of P-selectin and ICAM-1 in neutrophil infiltration during hepatic IRI. Liver damage was quantified via measurement of ALT levels and examination of liver histopathology. ELISA was used to measure the plasma levels of chemokines TNF-alpha, IL-6, CXCL1 and CXCL2. It was concluded that reperfusion, following an ischemic phase of 90 minutes, lead to significant hepatocellular injury in both wild type and P/I knockout mice. This suggested that the cellular adhesion molecules, P-selectin and ICAM-1, appear to not be critical for neutrophil-mediated hepatic IRI. P-selectin is not present in the sinusoidal epithelium of the liver, which may be a reason that no significant difference was observed between wild type and P/I knockout mice. A potential limitation to this experiment is that, although unlikely, the P/I knockout mice may not have been true I-CAM1 knockout mice as they may have had low levels of alternatively spliced forms of I-CAM1. Furthermore, even with the lack of I-CAM1, other cellular adhesion molecules, such as VCAM-1, were likely still functioning to provide neutrophil extravasation signaling [32]. Future studies, using both ICAM-1 and VCAM-1 knockout mice, may provide further insight into the role of cellular adhesion molecules in neutrophil-mediated hepatic IRI.

Monson et al. observed a statistically significant difference in chemokine levels between the wild type and P/I knockout mice. CXCL1 and CXCL2, chemokines that are activated in inflammatory states, were found to be significantly elevated in wild type mice in comparison to P/I knockout mice. This suggests that P-selectin and ICAM-1 may play a role in upregulating chemokine production, which provides a survival advantage for neutrophils. This study also showed that chemokine levels correlated with neutrophil infiltration and liver injury six hours after reperfusion. In contrast, at fifteen hours after reperfusion, chemokine levels had returned to baseline whereas neutrophil infiltration was at its peak [32]. In another study, Colletti et al. found that hepatic IRI led to increased production of epithelial neutrophil-activating protein (CXCL5), which led to subsequent increased neutrophil infiltration. They discovered this by neutralizing CXCL5, via passive immunization, and observed a significantly decreased neutrophil sequestration in the liver following IRI [32,33]. This suggests that the CXCL5 serves as a necessary chemokine for neutrophil trafficking in IRI. Lentsch et al. investigated the role of chemokines KC (CXCL1) and MIP-2 (CXCL2) in the induction of liver IRI. It was found that neutralization of CXCL1 and CXCL2 resulted in significantly decreased hepatic neutrophil accumulation and hepatocellular injury following IRI. This suggests that CXCL1 and CXCL2 serve an essential role in the signaling for neutrophil trafficking and migration into reperfused hepatic tissue following a period of ischemia [5,34]. In addition, Junction Adhesion Molecule A (CD321) is reported to be a crucial molecule for neutrophil trans-endothelial migration at the site of injury. CD321 is highly expressed at intercellular junctions, and more specifically at tight junctions in endothelial and epithelial cells [35]. A recent study has shown promising results for monoclonal antibody therapy against cell surface antigens as a potential preventative treatment against the damaging effects of neutrophil-induced liver IRI. Yin et al. examined the effect of anti-CD321 monoclonal antibody (mAb) administration on liver damage in a murine model after undergoing liver IRI. It was hypothesized that blunting its expression should decrease neutrophil infiltration after liver IRI. Their study found that administration of anti-CD321 mAb significantly reduced neutrophil infiltration early on after reperfusion. Decreased neutrophil infiltration significantly reduced liver IRI, which was quantified by aspartate aminotransferase and alanine aminotransferase levels in the serum at set time periods after reperfusion. Results also showed that there was decreased hepatocyte necrosis and apoptosis, and decreased upregulation of TNF-alpha and IL-6 in the mice that received anti-CD321 mAb after liver IRI. Therefore, the blockade of CD321 may serve as a potential therapy in reducing the inflammation and liver injury seen after liver IRI [36].

Similarly, the human microbiota has been an area of focus in the scientific community regarding its composition and effects on overall health of the host organism. The microbiota has been shown to play a fundamental role in regulation of the immune system. The variations in microbial composition of the microbiota between organism influences neutrophil production and function in different ways. Specific microbiota metabolites that influence neutrophil function are short-chain fatty acids, secondary bile acids, tryptophan metabolites and amines. Some microbial components upregulate neutrophil function, and others suppress it. Therefore, the differing specific microbial compositions of the microbiota may be a reason why clinical presentations of specific inflammatory diseases may vary from organism to organism. A delicate relationship exists between neutrophils and the microbiota in a normal steady state. The microbiota suppresses neutrophil recruitment and prevents an inflammatory response to their presence, whereas neutrophils secrete antimicrobial peptides to tightly regulate the composition of the microbiota [37]. Without this delicate balance between neutrophils and the microbiota, changes from the steady state can ensue. The microbiota is believed to serve an important role in liver IRI after liver transplant, via altering expression of host inflammatory cells. With this in mind, Ito et al. hypothesized that administration of rifaximin prior to liver transplant would lead to a reduction of harmful bacterial growth in the gut, and therefore reduce hepatic IRI. Researchers retrospectively examined liver transplant patients and grouped patients into either a rifaximin or control arm based on whether or not they received rifaximin prior to transplant. Results showed patients that received continuous rifaximin therapy for at least 28 days before surgery had lower rates of early allograft dysfunction and decreased serum levels of alanine aminotransferase and aspartate aminotransferase in comparison to the control group. Other studies have shown rifaximin to have a direct effect on bacterial growth and alter the composition of the microbiota, leading to decreased inflammation. Therefore, the mechanism behind the protective effect of rifaximin in liver transplant patients is likely its suppression of microbiota-induced neutrophil activation and subsequent inflammation [38].

## 6. Neutrophil Extracellular Traps as a Mediator of IRI

As previously mentioned, neutrophils play an important role in initiation and maintenance of liver IRI. Neutrophils maintain the pro-inflammatory state by production of proteolytic enzymes, arachidonic acid metabolites and ROS. Several metabolites and DAMPs can promote the activation and formation of neutrophil extracellular traps (NETs) in neutrophils. Activated neutrophils release MPO and other proteases that facilitate the formation of superoxide causing direct damage of liver endothelial cells. In addition, NETs which are DNA-linked, web-like structures can further facilitate damage by crosslinking with platelets to increase thrombosis, activation of immune cells or the complement system [39,40].

The exposure of intracellular proteins to the extracellular space in NETosis leads to potential autoantigen presentation to the host immune system [41]. This is thought to increase the release of DAMPs, which further activate the immune system and contribute to the inflammation of IRI.

NETs have also been seen to play a role in clotting and thrombus formation. In a murine model of liver IRI, our laboratory has shown that induction of NETs can further activate platelets results in systemic immune-thrombosis and distant organ injury [42,43]. This study showed that 1 h of ischemia followed by 6 h of reperfusion lead to activation of platelets and increased neutrophil platelet aggregation. These aggregates formed micro-thrombi within the liver following IRI. When NETs were blocked using DNAse-1 therapy, immunological thrombi and organ damage were significantly reduced. Interestingly, when utilizing platelet-specific TLR4 KO animals, we found significant decreased organ damage, with lower circulating platelet activation and platelet-neutrophil aggregates following liver I/R. This effect of TLR4-dependent platelet activation is thought to be mediated through HMGB1 released by neutrophils [43]. Similar results were found by Gould et al. showing that this effect indeed was depending on platelet expression of TLR2 and TLR4, suggesting that NET components may act as a receptor agonist promoting platelet activation.

**Figure 2:**
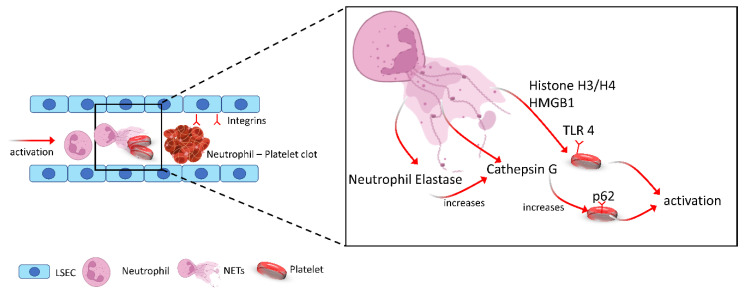
Schematic illustration showing increase in the release of NET-decorated proteins such as neutrophil elastase, cathepsin G, HMGB1 and histone upon NETosis. These proteins induce platelet activation through TLR4 and p62 pathways, resulting in the formation of microthrombi within the liver and promote IR induce liver injury.

Fuchs et al. have furthermore demonstrated the role of DNA as well as histones H3 and H4 in NET-induced platelet aggregation in vitro [44]. However, in their study, they found that NET-dependent platelet aggregation was shown to be unaffected by DNAse or heparin treatment, which would suggest a mechanism independent of the DNA structure and thrombin. They furthermore showed that blocking of cathepsin G, which is contained within the NET chromatin, led to reduced expression of CD62 and phosphatidylserine and decreased aggregation. This finding was further supported by Nemmar et al. who showed that neutrophil-specific elastase increased the cathepsin G-induced platelet aggregation mediated by neutrophils [45]. Similarly, NETs have also been shown to have direct thrombin inducing abilities in platelets, which was confirmed using PMA- treated neutrophils and platelet cocultures and this effect was abrogated using DNAse [46]. In addition to NET production, PMA-treated neutrophils have been shown to release microparticles that can attach themselves to NETs via phosphatidylserine residues. Blocking this interaction reduced the NET-dependent thrombin generation showing an important role of microparticles besides DNA and NETs in platelet activation and clot formation [46]. An overview is provided in Figure 2.

## 7. Neutrophil-Targeted Therapies

A number of studies have concentrated on neutrophils pro-inflammatory and tissue-destructive actions, as they have long been recognized as significant players in promoting liver I/R injury. Among many others, Formyl peptide, a DAMP released from mitochondria during stress, has shown to exert a chemotactic signaling for neutrophils via the FRP1 receptor. Masaki et al. investigated dynamic neutrophil recruitment in a murine model where a group of mice were subjected to partial warm hepatic I/R. In their model, mice were pretreated with an FPR1 antagonist, cyclosporine H (CsH), or formyl peptide, fMLF. They observed an alleviated hepatic I/R injury when treated with CsH, as evidenced by decreased serum transaminase levels, reduced hepatocyte necrosis/apoptosis, and diminished inflammatory cytokine, chemokine and oxidative stress [47].

In tandem with FPR1, we have previously reported that liver IRI upregulates the release and expression of IL-33 from stressed liver sinusoidal endothelial cells, which is recognized by the neutrophil ST2 receptor and promotes chemotaxis, activation and NET formation into the inflamed site. Similar results were observed in patients that underwent liver hepatectomies. Blocking IL-33 or ST2 by utilizing specific KO mice and subjecting them to warm liver IRI attenuated increased neutrophil influx and NET formation. Furthermore, adaptive transfer of neutrophils from ST2 KO mice into neutrophil-depleted WT recipients significantly decreased NET formation. In contrast, a recent study by Wang et al. showed that IL-33 promotes a significant increase in the percent of infiltrating eosinophil in liver and play a hepatoprotective role against IRI. In addition, adoptive transfer of eosinophils from the bone marrow improved liver damage in eosinophil-deficient mice and decreased hepatic ischemia and reperfusion injury in WT recipients [48].

NETs are the major byproduct of infiltrating neutrophils and are mostly present in the sinusoids of ischemic liver lobes. We have reported that treatment with peptidyl-arginine-deiminase 4 inhibitor or DNase I inhibits NET formation and significantly protects hepatocytes with reduced inflammation after liver I/R. Inhibition of NET formation by the peptidyl-arginine-deiminase 4 inhibitor and that DNase I decreases High Mobility Group Box 1 and histone-mediated liver I/R injury by Toll-like receptor (TLR4)- and TLR9-MyD88 signaling pathways [49].

Anding et al. demonstrated that fibrin-derived peptide Bβ15-42 attenuates liver damage in a rat model of liver ischemia/reperfusion injury. Bβ15-42 treatment decreased leukocyte infiltration and expression of hepatic inflammatory cytokines. Moreover, Bβ15-42 significantly reduced high-mobility group box 1 release and altered mitogen-activated protein kinase activation [50].

BTKB66 is a selective, irreversible inhibitor of Bruton tyrosine kinase (Btk). Tiziana et al. show that BTKB66 potently inhibited lipopolysaccharide-mediated activation of bone marrow-derived neutrophils and macrophages in vitro. It also reduced the severity of I/R injury as determined by AST and ALT levels, as well as immune cell infiltrates. BTKB66 significantly decreased hepatic markers of sterile inflammation, such as C-X-C motif chemokine 1, C-X-C motif chemokine 2 and C-X-C motif chemokine 10, in parallel with depression of serum markers of the myeloid cell activation, such as CCL5, CCL11 and C-X-C motif chemokine 5 [51].

Most recently, Huang et al. show that the IV Prussian Blue (PB) Scavenger was mainly distributed in the liver, where it showed excellent ability to alleviate apoptosis, tissue injury and organ dysfunction after HIRI. The PB scavenger was found to protect liver tissue by scavenging ROS, reducing neutrophil infiltration and promoting macrophage M2 polarization [52].

Jing et al. present an innovative strategy by integrating a platinum nanoantioxidant and inducible nitric oxide synthase into the zeolitic imidazolate framework-8 based hybrid nanoreactor for effective prevention of ischemia-reperfusion injury. They show that a platinum nanoantioxidant can scavenge excessive reactive oxygen species at the injury site and meanwhile generate oxygen for subsequent synthesis of nitric oxide under the catalysis of nitric oxide synthase. Such cascade reaction successfully achieves dual protection for the liver through reactive oxygen species clearance and nitric oxide regulation, enabling reduction of oxidative stress, inhibition of macrophage activation and neutrophil recruitment and ensuring suppression of proinflammatory cytokines [53].

## 8. Liver IRI and the Role of Small Non-Coding RNAs

In the recent literature, the role of non-coding RNAs in IRI has been investigated. Non-coding RNAs include both microRNA (miRNA) and long non-coding RNAs (lncRNAs). miRNA are short segments of RNA that regulate transcription of genes that are involved in cell survival, responses to stress and apoptosis. miRNA have historically been known to play roles in both innate and adaptive immunity but its role in IRI has been unknown until recently. They have been found to play a role in IRI via modification of cellular pathways involved in inflammation, necrosis and apoptosis. The role of different miRNAs has been assessed in many tissues, including the liver, kidney, intestine, heart and brain [54]. The expression of specific miRNAs were found to be altered by IRI in these tissues, as IRI causes either upregulation or downregulation of miRNA during IRI in comparison to their normal baseline level of expression. Specific miRNAs, miR-210 and miR-155, have been found to be upregulated during liver IRI in mouse models. In hepatocytes, mIR-210 may potentially play a role in hepatic IRI via formation of a negative feedback loop with SMAD4. mIR-155 has been shown to activate the NF-KB pathway, which may lead to an exaggerated inflammatory response during IRI [55]. mIR-155 also suppresses SOCS1, which may further potentiate hepatic IRI [56]. A recent study by Tan et al. showed that miR-155 aggravates liver IRI via downregulation of SOCS1 protein expression. It was hypothesized that miR-155 overexpression promotes liver IRI via SOCS1 inhibition. Lack of SOCS1 leads to increased release of the proinflammatory cytokines, IL-6 and TNF-alpha, via activation of NF-KB. This hypothesis was tested as one group of mice was transfected with Ago-miR-155 via tail vein injection, then underwent hepatic IRI 24 hours later. SOCS1 expression was then measured and compared between the experimental and wild type groups of mice. Ago-miR-155-injected mice were found to have significantly decreased amounts of SOCS1 protein, and resulting enhanced liver inflammation after liver IRI, in comparison to wild type mice [57]. These results support the hypothesis that increased miR-155 expression leads to hepatic inflammation in IRI via downregulation of SOCS1. Therefore, miR-155 inhibition may serve as a promising therapy in the reduction of liver IRI.

In contrast, some miRNA subtypes are downregulated when liver IRI occurs. miR-122, miR-30b, miR-20a, miR-125b, miR-494, miR-148a and miR-93 have all been shown to be decreased in liver IRI. Therefore, an increase in these specific miRNAs may serve as a protective role against IRI in the liver specifically. Upregulation of miR-125b inhibits the NF-KB pathway, which prevents the release of proinflammatory cytokines and could protect against liver IRI. miR-494 functions to activate the PI3K/AKT signaling pathway, and also inhibits TRAF6, which could both lead to attenuation of liver IRI. miR-148a dephosphorylates TAK1 and IRF3, which are activators of TLR4 signaling, a known promoter of inflammation in liver IRI. Therefore, miR-148a upregulation may provide protection against liver IRI. miR-93 may decrease hepatic damage in liver IRI via inhibition of STAT3, a known regulator of inflammation [56]. Overall, some specific miRNA subtypes serve to protect against liver IRI and others play a role in promoting inflammation and damage in liver IRI. Future therapies to modulate the expression of certain miRNA subtypes may be beneficial in protecting the liver from IRI.

## 9. Conclusions

This review discusses the role of different signaling pathways and cell types that aid in the induction or maintenance of IRI. DAMPs are released from necrotic cells that can further promote the activation of pro-inflammatory cascades. Neutrophils are among the first cells that are at the liver following IRI. In circulation, neutrophils can activate platelets and form immunologic microthrombi that can worsen ischemia. Within the tissue, they are producers of ROS and can release NETs upon activation. Taken together, this review provides an overview of different signaling pathways and cells that participate in IRI.

## Data Availability

Not applicable.
